# *Agaricus blazei* Extract Induces Apoptosis through ROS-Dependent JNK Activation Involving the Mitochondrial Pathway and Suppression of Constitutive NF-**κ**B in THP-1 Cells

**DOI:** 10.1093/ecam/nep176

**Published:** 2011-02-20

**Authors:** Mun-Ock Kim, Dong-Oh Moon, Jin Myung Jung, Won Sup Lee, Yung Hyun Choi, Gi-Young Kim

**Affiliations:** ^1^Laboratory of Immunobiology, Department of Marine Life Science, Jeju National University, Jeju, Republic of Korea; ^2^Department of Neurosurgery, Institute of Health Science, Gyeongsang National University School of Medicine, Republic of Korea; ^3^Department of Internal Medicine, Institute of Health Science, Gyeongnam Regional Cancer Center, Gyeongsang National University School of Medicine, Jinju, Republic of Korea; ^4^Department of Biochemistry, Dongeui University College of Oriental Medicine, Busan, Republic of Korea

## Abstract

*Agaricus blazei* is widely accepted as a traditional medicinal mushroom, and it has been known to exhibit immunostimulatory and anti-cancer activity. However, the apoptotic mechanism in cancer cells is poorly understood. In this study, we have investigated whether *A. blazei* extract (ABE) exerts antiproliferative and apoptotic effects in human leukemic THP-1 cells. We observed that ABE-induced apoptosis is associated with the mitochondrial pathway, which is mediated by reactive oxygen species (ROS) generation and prolonged c-Jun N-terminal kinase (JNK) activation. In addition, the ABE treatment resulted in the accumulation of cytochrome *c* in the cytoplasm, an increase in caspase activity, and an upregulation of Bax and Bad. With those results in mind, we found that ABE decreases constitutive NF-**κ**B activation and NF-**κ**B-regulated gene products such as IAP-1 and -2. We concluded that ABE induces apoptosis with ROS-dependent JNK activation and constitutive activated NF-**κ**B inhibition in THP-1 cells.

## 1. Introduction

Reactive oxygen species (ROS), such as hydrogen peroxides (H_2_O_2_), hydroxyl radials (^•^OH), and superoxide anions (O_2_^−^), are normally generated by the mitochondria and other intracellular molecules in all aerobes [1]. In general, the redox status is well balanced by the enzyme and non-enzyme systems in cells. Oxidative stress occurs when the critical balance is disrupted by excess ROS production and/or antioxidant depletion [2]. Many chemotherapeutic agents may be selectively toxic to tumor cells because they increase oxidant stress [3–5]. Increased intracellular ROS levels could trigger apoptosis by the activation of the mitochondrial-dependent cell death pathway through the activation of mitogen-activated protein kinase (MAPK) pathways and the proapoptotic Bcl-2 proteins Bax or Bad, thus subsequently inducing mitochondrial membrane permeabilization and cell death [4–6].

Nuclear factor-kappa B (NF-*κ*B) is a prominent factor in cell death or survival. Optimal activation of NF-*κ*B requires phosphorylation of the NF-*κ*B p65 protein by IKK, along with the activation of a variety of other kinases [7, 8]. Among the target genes, the inhibitors of apoptosis proteins (IAPs) are believed to be critical cell survival signals. Previous studies have reported that the IAPs bind to caspases and inhibit apoptosis [9], indicating that down-regulation of these proteins and the subsequent activation of caspases is one of the primary mechanisms of apoptosis in various types of cancer cells [10, 11]. Because NF-*κ*B can promote cell survival and reduction in its activity leads to apoptosis [12–14], NF-*κ*B-suppressing agents may be potentially useful in the prevention and treatment of cancer.

Many natural dietary extracts from mushrooms have been found to be potent in the treatment of various cancers [15, 16]. Mixed mushroom extracts might contain different chemotherapeutic properties with more than one mechanism of action, thus possessing a combination of different chemotherapeutic effects. Therefore, it is very important to identify the combined effects from mixed mushroom extracts. The basidiomycete mushroom *Agaricus blazei* has been traditionally used in health foods as a source for the prevention of cancer, hyperlipidemia, arteriosclerosis, and chronic hepatitis. *Agaricus blazei* extract (ABE) has been reported to have anti-tumor and anti-mutagenic effects [17–19]. The intratumoral [20] or oral administration [21] of ABE also resulted in the infiltration of natural killer (NK) cells in the tumor sites and increased NK cell activity in animals. Our previous studies also demonstrated that ABE induces the phenotypic and functional maturation of murine bone marrow-derived dendritic cells [22], and it increases apoptosis through Akt dephosphorylation in human leukemic U937 and G_2_/M cell cycle arrest in human gastric epithelial AGS cells, respectively [23, 24]. Although numerous studies on the immunostimulatory effects of ABE have been reported, the cellular and molecular mechanisms underlying ABE-induced ROS generation and apoptosis are not clear.

In this study, to further elucidate the abilities of ABE-induced apoptosis combined with ROS production, we investigated the effect of ABE on Jun N-terminal kinase (JNK) activation and NF-*κ*B inhibition in human leukemic THP-1 cells. We report here that ABE increases the intracellular ROS level in a time-dependent manner, and the increase is responsible for its apoptotic effects. ABE-induced ROS activated JNK, leading to mitochondria-dependent apoptosis. ABE treatment markedly resulted in the accumulation of cytochrome *c* in the cytoplasm, an increase in caspase activity, and an upregulation of Bax and Bad. With those results in mind, we found that ABE suppressed constitutive NF-*κ*B activation and NF-*κ*B-regulated gene expression in THP-1 cells. Finally, the ABE-mediated ROS generation closely correlates with the onset of apoptosis.

## 2. Methods

### 2.1. Reagents

Propidium iodide (PI), paraformaldehyde, 3-(4,5-dimethyl-2-thiazolyl)-2,5-diphnyl-2*H*-tetrazolim bromide (MTT), and *N*-acetyl-cysteine (NAC) were purchased from Sigma (St. Louis, MO). 3,3-Dihexyloxacarbocyanine iodide (DiOC_6_), 2′,7′-dichlorofluorescein diacetate (DCFDA), and hydroethidine (HE) were purchased from Molecular Probes (Eugene, OR). SP600125 were obtained from Calbiochem (San Diego, CA). RPMI 1640 medium and fetal bovine serum (FBS) were purchased from Invitrogen Co. (Carlsbad, CA) and Gibco BRL (Gaithersburg, MD), respectively. All other chemicals not specifically cited here were purchased from Sigma. ABE was prepared as previously described [23] and was supplied from Daejeon University Oriental Hospital (Daejeon, Republic of Korea).

### 2.2. Antibodies

Antibodies for IAP-1, IAP-2, Bax, Bad, Bcl-2, PARP, caspase-3, caspase-8, caspase-9, p65, p50 and nucleolin were purchased from Santa Cruz Biotechnology (Santa Cruz, CA). *β*-Actin antibody was purchased from Sigma and antibodies for JNK and phospho (p)-JNK were purchased from Cell Signaling (Beverly, MA). Cytochrome *c* antibody was purchased Chemicon International (Temecula, CA).

### 2.3. Cell Culture and Viability Assay

Human leukemic THP-1 cells were obtained from the American Type Culture Collection (Manassas, VA) and maintained in RPMI 1640 culture medium containing 10% heat-inactivated FBS and 1% penicillin-streptomycin (Sigma). Cells were seeded at 2 × 10^5^ cells ml^−1^, treated with ABE, and then incubated for the indicated time. Cell viability was determined by MTT assay.

### 2.4. Flow Cytometric Analysis

Cells were fixed in 70% ethanol overnight at 4°C. The cells were washed in phosphate-buffered saline (PBS) with 0.1% BSA. Cells were incubated with 1 U ml^−1^ of RNase A (DNase free) and 10 *μ*g ml^−1^ of PI overnight at room temperature in the dark. Cells were analyzed using a FACSCalibur flow cytometer (Becton Dickenson, San Jose, CA), and CellQuest software (Becton Dickinson) was used to determine the relative DNA content based on the presence of a red fluorescence.

### 2.5. Determination of Mitochondrial Membrane Potential

Mitochondrial membrane potential was monitored by measuring the uptake of DiOC_6_. Briefly, cells were harvested, loaded with 50 nM DiOC_6_ at 37°C for 30 min in the dark, and then analyzed using a flow cytometer.

### 2.6. *In Vitro* Caspase Activity Assay

Activity of caspase-like protease was measured using a caspase activation kit according to the manufacturer's protocol (R&D systems; Minneapolis, MN). This assay is based on spectrophotometric detection of the color reporter molecule *p*-nitroaniline (*p*NA), which is linked to the end of the caspase-specific substrate. The caspase cleaves the peptide and releases the chromophore *p*NA, which can be quantified spectrophotometrically at a wavelength of 405 nm.

### 2.7. Western Blot Analysis

Total cell extracts were prepared using a PRO-PREP protein extraction solution (iNtRON Biotechnology; Sungnam, Republic of Korea). For assay of cytochrome *c* release, mitochondrial fractions were prepared using a mitochondria isolation kit for mammalian cells (Pierce; Rockford, IL) according to the manufacturer's instructions. The preparation of cytoplasmic and nuclear extracts was conducted using the NE-PER nuclear and cytoplasmic extraction reagents (Pierce). Cell extracts were separated on 8 or 10% polyacrylamide gels (PAGE), and then transferred to nitrocellulose membranes using standard procedures. The membranes were developed using an ECL reagent (Amersham; Arlington Heights, IL).

### 2.8. ROS Measurement

Generation of intracellular ROS was examined by flow cytometry using DCFDA for H_2_O_2_ and HE for O_2_^−^. The cells were exposed to 4 mg ml^−1^ ABE and/or 10 mM NAC for various times, and then treated with 10 *μ*M DCFDA and 2 *μ*M HE for 30 min at 37°C. Subsequently, the cells were collected, washed twice with PBS, and analyzed for DCFDA and HE fluorescence using a flow cytometer.

### 2.9. Electrophoretic Mobility Shift Assays (EMSA)

DNA-protein binding assays were carried out with a nuclear extract. Synthetic complementary NF-*κ*B (5′-AGT TGA **GGG GAC TTT CCC** AGG C-3′) binding oligonucleotides (Santa Cruz Biotechnology) were 3′-biotinylated using the biotin 3′-end DNA labeling kit (Pierce) according to the manufacturer's instructions.

### 2.10. Statistical Analysis

All data were derived from at least three independent experiments. The blots were visualized with Chemi-Smart 2000 (Vilber Lourmat, Marine; Cedex, France). Images were captured using Chemi-Capt (Vilber Lourmat) and transported into Photoshop. Statistical analyses were conducted using SigmaPlot software and values were presented as mean ± SD. Significant differences between the groups were determined using the unpaired Student's *t*-test. A value of *P < .05 was accepted as an indication of statistical significance.

## 3. Result

### 3.1. ABE Decreases Cell Viability and Induces Apoptosis in THP-1 Cells

**Figure 1:**
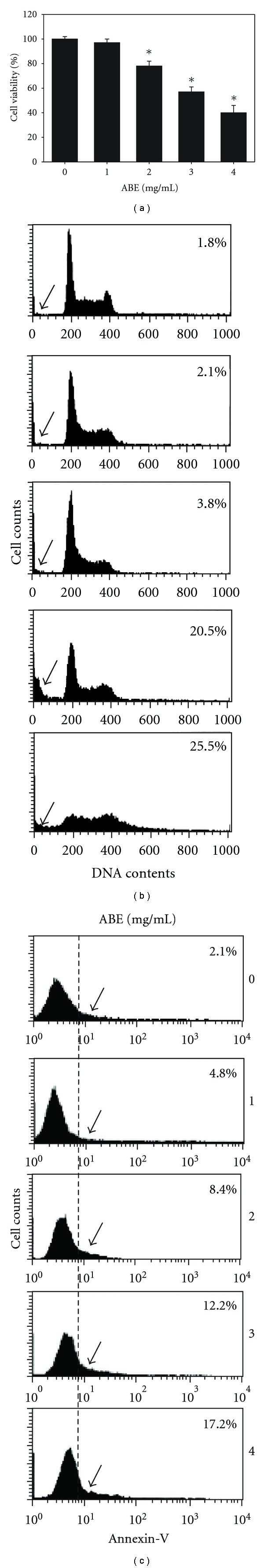
Effect of ABE on cell viability and cell death in THP-1 cells. THP-1 cells were seeded at 2 × 10^5^ cells ml^−1^ and were then treated with ABE at the indicated concentrations for 24 h. (a) Cell viability was determined by MTT assay. Each point represents the mean ± SD of three independent experiments. The significance was determined by Student's *t*-test (*P < .05 versus vehicle control (0)). (b) DNA contents and (c) annexin-V^+^ cells were analyzed by flow cytometer. Percentages represents the sub-G_0_/G_1_ phase and annexin-V^+^ cells, respectively. The results shown are from one representative experiment of three experiments that exhibited similar patterns.

To determine the effect of ABE on cell viability, human leukemic THP-1 cells were treated with increasing concentrations up to 4 mg ml^−1^ and incubated for 24 h. Cell viability was examined using MTT assays. As shown in Figure 1(a), ABE treatment resulted in a dose-dependent inhibition of viability in THP-1 cells. Indeed, to elucidate the ability of ABE in its anti-viable activity, we analyzed cell cycle distribution using flow cytometry. As shown in Figure 1(b), ABE caused a significant increase in the fraction of sub-G_0_/G_1_ phase in a dose-dependent manner. For example, as compared with control, the percentage of cells in sub-G_0_/G_1_ phase was increased more than 10-fold upon treatment with 4 mg ml^−1^ ABE for 24 h. Analysis of cell cycle distribution suggests that ABE treatment might cause apoptosis in THP-1 cells. Therefore, we further examined the apoptosis-inducing effect of ABE by annexin-V analysis. As shown in Figure 1(c), ABE treatment caused significant increases in apoptotic cell percentages as compared with control cells (17% of apoptotic cells in cells with 4 mg ml^−1^ ABE for 24 h). Therefore, we observed that the proliferation of THP-1 cells was significantly suppressed with an increase of apoptotic percentage in the presence of ABE.

### 3.2. Mitochondria-Dependent Pathways Are Involved in ABE-Induced Apoptosis

**Figure 2:**
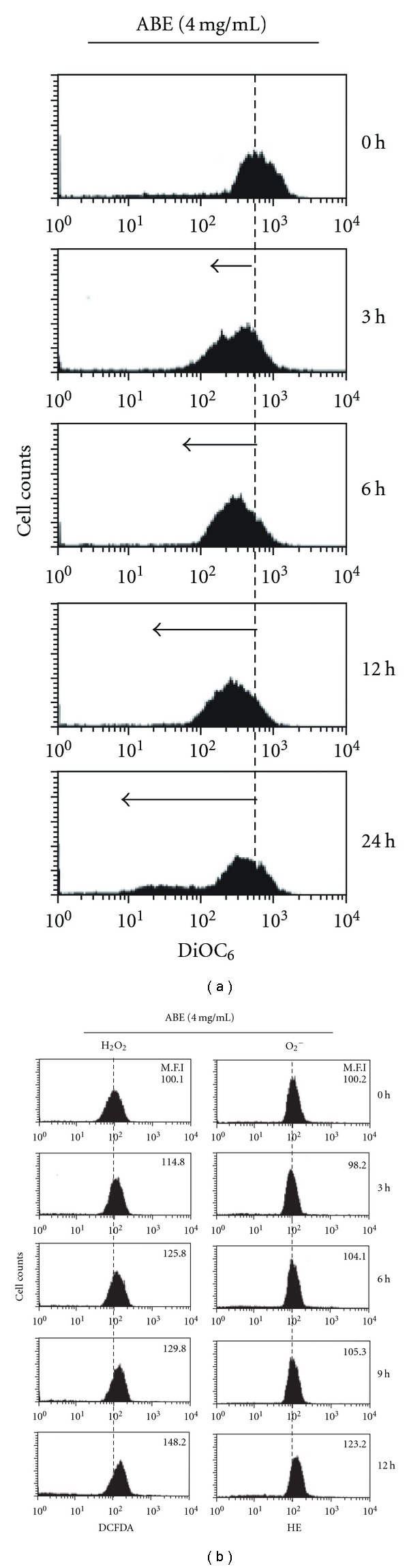
Effect of ABE on mitochondrial function and ROS generation. THP-1 cells were seeded at 2 × 10^5^ cells ml^−1^ and were then treated with 4 mg ml^−1^ of ABE for the indicated time. (a) The mitochondrial membrane potential was determined by flow cytometer using DiOC_6_ labeling of mitochondria. (b) The redox status was monitored by the oxidation-sensitive fluorescent dyes for H_2_O_2_ (DCFDA) and O_2_^−^ (HE). The results shown are from one representative experiment of three experiments that showed exhibited similar patterns.

To further characterize whether ABE induces apoptosis by triggering the mitochondrial apoptosis pathway, we investigated the depolarization of the mitochondrial membrane potential and the generation of ROS. When the THP-1 cells were exposed to 4 mg ml^−1^ ABE up to 24 h, the mitochondrial membrane potential was significantly reduced in a time-dependent manner as evidenced by an increase in the proportion of cells with lower fluorescence intensity (Figure 2(a)). As shown in Figure 2(b), treatment with 4 mg ml^−1^ ABE also significantly increased the intensity of DCFDA and HE fluorescence (indicating quantification of peroxides and superoxides, resp.).

**Figure 3:**
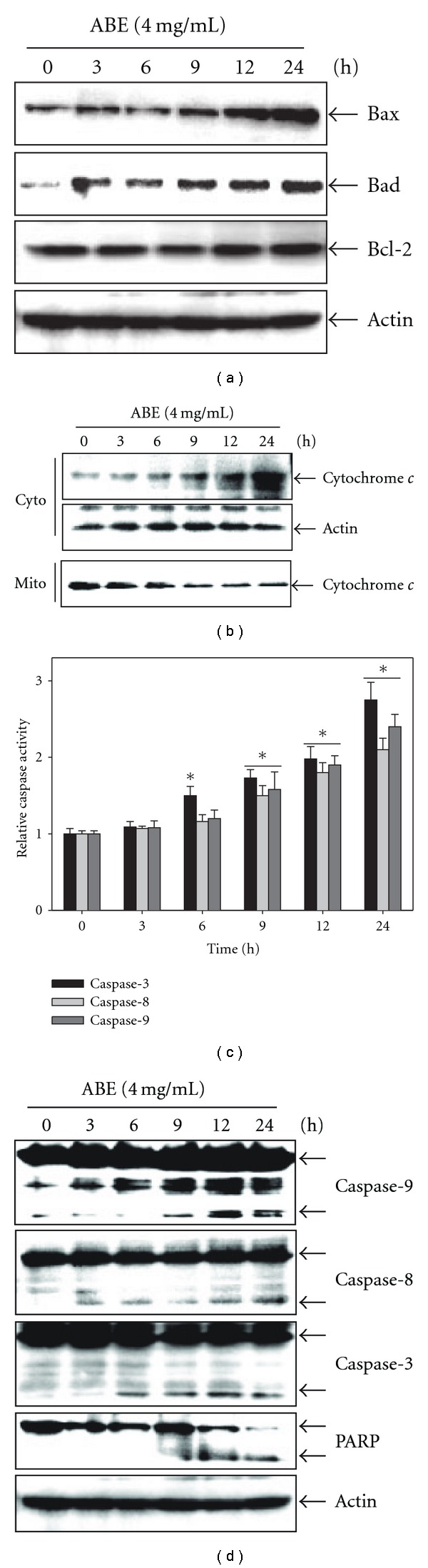
Effect of ABE on expression of apoptotic proteins. THP-1 cells were seeded at 2 × 10^5^ cells ml^−1^ and were then treated with 4 mg ml^−1^ of ABE for the indicated time. (a) Total cell extracts resolved by SDS–PAGE, transferred to nitrocellulose, and probed with specific antibodies (a) anti-Bax, anti-Bad and anti-Bcl-2; (d) anti-caspase-3, anti-caspase-8, anti-caspase-9 and anti-PARP). (b) Equal amounts of mitochondria and cytosol cell lysates were resolved by SDS–PAGE, transferred to nitrocellulose, and probed with a specific antibody for cytochrome *c*. Equal protein loading was evaluated by *β*-actin. The results shown are from one representative experiment of three experiments that exhibited similar patterns. (c) Caspase (-3, -8 and -9) activities were determined using caspase assay kits. Each point represents the mean ± SD of three independent experiments. The significance was determined by Student's *t*-test (*P < .05 versus vehicle control (0)). Cyto, Cytoplasm; Mito, Mitochondria.

We next monitored the activation of caspases and the expression of the pro- and anti-apoptotic proteins. Western blot analysis showed that treatment with 4 mg ml^−1^ ABE significantly increased pro-apoptotic proteins Bax and Bad, and showed the steady presence of the anti-apoptotic protein Bcl-2 over the course of time (Figure 3(a)). In addition, ABE caused cytochrome *c* to be released from the mitochondria into the cytosol in a time-dependent manner (Figure 3(b)). We also examined the effect of ABE on caspase activity, as well as cleavage of caspases and PARP in THP-1 cells treated with 4 mg ml^−1^ of ABE. As expected, caspase-9 activity was significantly increased (Figure 3(c)), as ABE induces cytochrome *c* release into the cytosol (Figure 3(b)). Our results were also showed increased activation of caspase-8 and -3 (Figure 3(c)). As shown in Figure 3(d), the cleavages of caspase and PARP revealed in a time-dependent manner. These finding indicate that ABE-induced apoptosis was mediated with the mitochondrial pathway.

### 3.3. Inhibition of ROS Generation Blocks ABE-Induced Apoptosis

**Figure 4:**
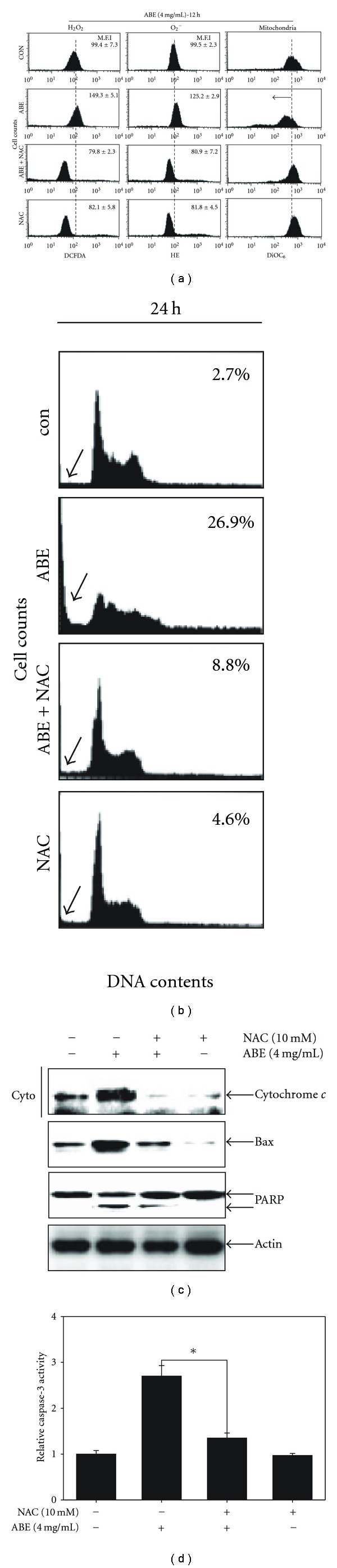
The role of ROS on ABE-mediated apoptosis. THP-1 cells (2 × 10^5^ cells ml^−1^) were pretreated with 10 mM NAC, and then 4 mg ml^−1^ of ABE was treated for 12 h (a) and 24 h (b–d). (a) The redox status was monitored by the oxidation-sensitive fluorescent dyes for O_2_^−^ and H_2_O_2_. The mitochondrial membrane potential was also determined by flow cytometer using DiOC_6_ labeling of mitochondria. (b) DNA contents were analyzed by flow cytometer. The sub-G_0_/G_1_ DNA fractions, indicating apoptosis, were determined as a percentage of the total number of cells. (c) Equal amounts of cell lysate were resolved on SDS-PAGE, transferred to nitrocellulose membranes, and probed with specific antibodies for cytochrome *c*, Bax, and PARP. *β*-Actin was used as an internal loading control. (d) THP-1 cells were collected following 24 h exposure to 4 mg ml^−1^ of ABE with or without 2 h of pretreatment with NAC. Caspase-3 activity was determined following the manufacturer's protocol. Each point represents the mean ± SD of three independent experiments. The significance was determined by Student's *t*-test (*P < .05 versus vehicle control). Cyto, Cytoplasm.

To investigate whether ROS generation is directly associated with ABE-induced mitochondria dysfunction and apoptosis, we assessed these events in THP-1 cells pretreated with NAC (10 mM) for 2 h followed by treatment with 4 mg ml^−1^ ABE. As expected, pretreatment with NAC decreased ROS generation and normalized mitochondrial dysfunction caused by ABE in THP-1 cells (Figure 4(a)). To next determine whether pretreatment with NAC could block ABE-induced apoptosis, we performed cell cycle analysis using flow cytometry. As shown in Figure 4(b), ABE caused a significant increase in the fraction of sub-G_0_/G_1_ phase, but NAC pretreatment reversed it. For example, as compared with control, the percentage of cells in sub-G_0_/G_1_ phase was increased than 10-fold upon treatment with 4 mg ml^−1^ ABE for 24 h, but only increased more than 3-fold upon pretreatment with NAC.  Figure 4(c) also shows that NAC decreased ABE-mediated Bax expression, cytochrome *c* release and PARP cleavage in ABE-treated THP-1 cells. In addition, the activation of caspase-3 induced by ABE was markedly attenuated by pretreatment with NAC (Figure 4(d)). These results suggest that the production of ROS plays an important role in ABE-mediated mitochondrial apoptotic pathways.

### 3.4. JNK Activation Is a Potential Target in ABE-Induced Apoptosis

**Figure 5:**
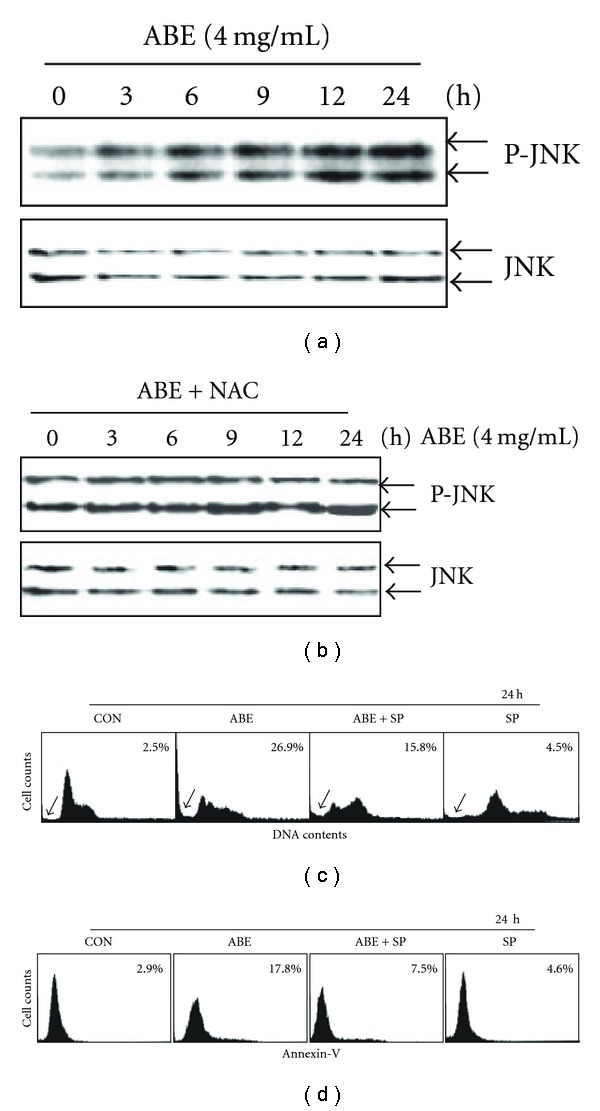
The role of JNK activation on ABE-mediated apoptosis. THP-1 cells (2 × 10^5^ cells ml^−1^) were treated with 4 mg ml^−1^ of ABE for the indicated time. (a) Equal amounts of whole cell lysates were resolved by SDS-PAGE, transferred to nitrocellulose and probed with specific antibodies against p-JNK and JNK. (b) THP-1 cells were pretreated with 10 mM NAC and then 4 mg ml^−1^ of ABE was treated up to 24 h. Then, the cells were lysated and western blot was performed using anti-p-JNK and anti-JNK antibodies. THP-1 cells were pretreated with SP600125 (20 *μ*M) for 2 h followed by ABE treatment (4 mg ml^−1^) for 24 h. At the end of experiments, (c) DNA content and (d) annexin-V were analyzed by flow cytometer. SP, SP600125.

As it has been shown that the ROS-mediated DNA damage triggers activation of JNK and subsequent cell death [4–6], we examined the status of JNK phosphorylation after ABE treatment. As shown in Figure 5(a), activation (phosphorylation) of JNK was significantly induced in a time-dependent manner and persisted for the duration of the experiment. On the other hand, the constitutive expression of JNK was not affected by ABE treatment. To further analyze whether ABE-induced ROS generation is responsible for JNK activation, cells were pretreated with NAC and then exposed to ABE up to 24 h. As shown in Figure 5(b), pretreatment with NAC prevented the increased phosphorylation of JNK caused by ABE. To further analyze whether activation of JNK is responsible for ABE-induced apoptosis, we exposed it to 20 *μ*M of SP600125, a JNK1/2 inhibitor for 2 h, followed by treatment with 4 mg ml^−1^ of ABE for 24 h. As shown in Figures 5(c) and 5(d), pretreatment of SP600125 significantly suppressed ABE-induced sub-G_0_/G_1_ cell population and annexin-V^+^ cells. These data confirm that treatment with ABE increases the production of ROS, leading to the activation of JNK.

### 3.5. ABE Inhibits Constitutive NF-*κ*B Activation and NF-*κ*B-Dependent IAPs

**Figure 6:**
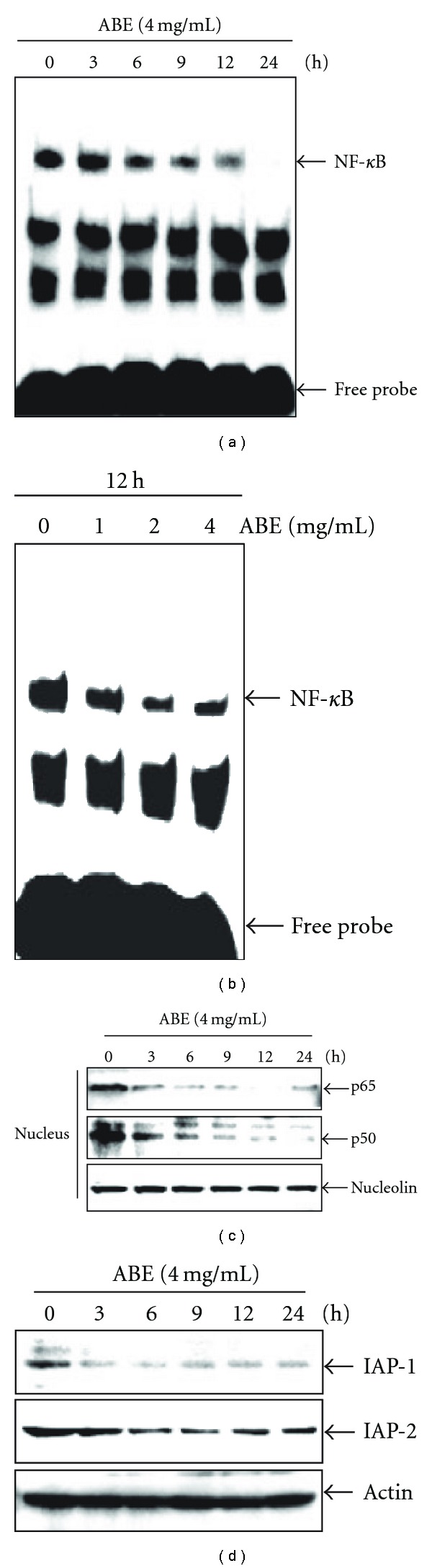
Effect of ABE on constitutive NF-*κ*B activation in THP-1 cells. THP-1 cells (2 × 10^5^ cells ml^−1^) were incubated at 37°C with (a) the indicated time duration of 4 mg ml^−1^ of ABE and (b) the indicated concentrations at 12 h. DNA-binding activity of NF-*κ*B was analyzed by lightShift^TM^ chemiluminescent EMSA kit. (c) Western blots were performed for p65 and p50 protein levels in the nuclear extract. Nucleolin was used as an internal loading control. (d) Expression of IAP-1 and -2 was detected via western blot analysis. *β*-Actin was used as an internal loading control for cytoplasm. The results shown are from one representative experiment of three experiments that exhibited similar patterns.

Because NF-*κ*B activation inhibits prolonged JNK activation and prevents the accumulation of ROS [25, 26], we examined the effect of ABE on the constitutive DNA-binding activity of NF-*κ*B and the NF-*κ*B-dependent responsive genes. First, THP-1 cells were treated with 4 mg ml^−1^ ABE over increasing time periods, and we then performed EMSA. As shown in Figure 6(a), treatment with ABE inhibited constitutive NF-*κ*B binding activity in a time-dependent manner. The level of NF-*κ*B inhibition at 24 h exposure of ABE was found to be almost completely inhibited. A similar inhibitory effect of ABE on constitutive NF-*κ*B binding activity at the 12 h time point was observed in a dose-dependent study (Figure 6(b)). Next, we confirmed the effect of ABE on constitutive NF-*κ*B activation in THP-1 cells by western blot analysis. As shown in Figure 6(c), ABE treatment resulted in a time-dependent decrease in protein levels of p65 and p50 in the nuclear extract.

Finally, we investigated whether ABE inhibits the NF-*κ*B-dependent transcriptional activated IAPs. Treatment with 4 mg ml^−1^ of ABE was examined for the expression of these genes via western blot analysis. As shown in Figure 6(d), treatment with ABE decreased the expressions of IAP-1 and -2 in a time-dependent manner, which is consistent with a decrease in NF-*κ*B activity. Therefore, it is possible that the induction of THP-1 cell apoptosis by ABE may be due to the inhibition of various anti-apoptotic factors.

## 4. Discussion

**Figure 7:**
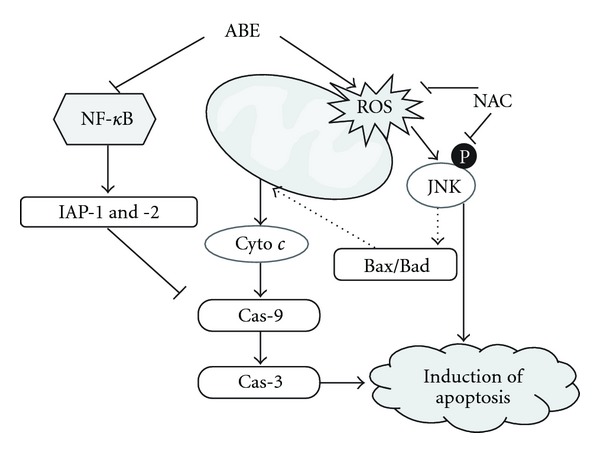
Proposed mechanisms for the apoptotic effects of ABE in THP-1 cells. The apoptotic mechanism was mediated by the generation of ROS, phosphorylation of JNK, release of cytochrome *c* into cytoplasm, and activation of caspases. Additionally, expression of IAPs, which have inhibitory effect of caspases, was abolished by inhibition of NF-*κ*B.

The present study reveals that ABE induced apoptosis through ROS-dependent JNK activation and inhibition of constitutively activated NF-*κ*B in human leukemic THP-1 cells. The ABE-induced ROS generation and activation of JNK that are associated with apoptotic cell death are significantly suppressed by pretreatment with NAC, indicating that ROS are the upstream-regulators of JNK activation during ABE-induced apoptosis. Based on the results of the present study, the mechanism by which ABE induces apoptosis in THP-1 cells is summarized in Figure 7.

Enhancement of ROS production has been associated with the apoptotic response induced by various chemotherapeutic agents [3–5]. ROS can cause apoptotic cell death via a variety of mechanisms, among which the activation of JNK plays a key role. It is identified by several reports indicating tumor necrosis factor-induced ROS production is responsible for sustained JNK activation in NF-*κ*B-activation deficient cells [25–27]. Here, we show that ROS production in ABE-treated human leukemic THP-1 cells is markedly increased and functionally linked to JNK activation during apoptosis via the mitochondrial-dependent pathway. We further observed that an antioxidant NAC can regulate ROS detoxification, which inhibits JNK activation and decreases apoptosis induced by ABE. These results suggest that ROS accumulation contributes to ABE-induced cell death.

JNK, a stress-activated protein kinase, is activated by many stress stimuli such as UV and ionizing irradiation, heat shock, cycloheximide, anti-cancer agents and pro-inflammatory cytokines, all of which can induce apoptosis [28, 29]. How activated JNK contributes to cell death is not clearly defined, but the production of tBid, a proteolytic fragment of Bid that specifically induces the release of mitochondrial Smac, points to JNK's role in JNK in promoting the initiation of the mitochondrial pathway of apoptosis [26]. Activated JNK has been also shown to phosphorylate Bcl-2 and Bcl-xL, thereby inactivating their anti-apoptotic functions [26]. Whereas previous studies presented that NF-*κ*B activation promotes cell survival by up-regulating the anti-apoptotic proteins' expression, including Bcl-2 as well as IAP families, and down-regulating the ROS-dependent JNK activation through suppression of the ROS accumulation [27, 30]. NF-*κ*B activation can also maintain tumor cell viability, and inhibiting NF-*κ*B activation alone can be sufficient to induce cell death [31, 32]. In addition, the involvement of NF-*κ*B in chemoresistance may be related to its constitutive activation, such as human acute myelogenous leukemic cells [33]. Therefore, we used human leukemic THP-1 cells in this experiment as a model. Several reports have shown that NF-*κ*B inhibitors act as potent enhancers of chemotherapy-induced apoptosis [34]. In agreement with previous findings, ABE significantly inhibited constitutive NF-*κ*B activation *in vitro*. NF-*κ*B-dependent IAP-1 and -2 were also suppressed in ABE-treated cells. These data indicate that ABE-induced NF-*κ*B inhibition enhances the apoptosis in ABE-treated cells, because decreased NF-*κ*B activity could not prevent ABE-induced ROS-dependent sustained JNK activation.

In conclusion, our data indicate that human leukemic THP-1 cells are highly sensitive to growth inhibition and apoptosis induction by ABE. ABE-induced apoptosis is associated with the mitochondrial pathway, and they are mediated by ROS generation and prolonged JNK activation. Taken together, ABE inhibits constitutive NF-*κ*B activity and NF-*κ*B-dependent IAP-1 and -2.

## Funding

National R&D Program for Cancer Control, Ministry of Health & Welfare, Republic of Korea (no. 8020050).
